# Understanding suicidal transitions in Australian adults: protocol for the LifeTrack prospective longitudinal cohort study

**DOI:** 10.1186/s12888-023-05335-1

**Published:** 2023-11-08

**Authors:** Philip J Batterham, Monica Gendi, Helen Christensen, Alison L. Calear, Fiona Shand, Matthew Sunderland, Rohan Borschmann, Michelle Banfield, Bridianne O’Dea, Mark Larsen, Cassandra Heffernan, Dominique Kazan, Aliza Werner-Seidler, Andrew J Mackinnon, Emily Hielscher, Jin Han, Katherine M Boydell, Liana Leach, Louise M Farrer

**Affiliations:** 1 Centre for Mental Health Research, The Australian National University, 63 Eggleston Road, Canberra, ACT 2601 Australia; 2 Black Dog Institute, University of New South Wales, Sydney, Australia; 3 The Matilda Centre for Research in Mental health and Substance use, University of Sydney, Sydney, Australia; 4 Melbourne School of Population and Global Health, Centre for Mental Health, The University of Melbourne, Melbourne, Australia; 5 Department of Psychiatry, Medical Sciences Division, University of Oxford, Oxford, UK; 6 Centre for Adolescent Health, Murdoch Children’s Research Institute, Melbourne, Australia; 7 School of Population Health, Curtin University, Perth, Australia; 8 Centre for Big Data Research in Health, University of New South Wales, Sydney, Australia; 9 School of Psychology, University of New South Wales, Sydney, Australia; 10 The Council of the Queensland Institute of Medical Research, Brisbane, Australia; 11 School of Public Health, Faculty of Medicine, The University of Queensland, Brisbane, Australia; 12 Flourish Australia, Sydney, Australia; 13 Center for Global Health Equity, New York University Shanghai, Shanghai, China; 14 National Centre for Epidemiology and Population Health, The Australian National University, Canberra, Australia

**Keywords:** Suicide, Attempt, Ideation, Transition, Australia, Cohort study

## Abstract

**Background:**

The factors that influence transition from suicidal ideation to a suicide attempt or remission of suicidal thoughts are poorly understood. Despite an abundance of research on risk factors for suicidal ideation, no large-scale longitudinal population-based studies have specifically recruited people with suicidal ideation to examine the mechanisms underlying critical transitions to either suicide attempt or recovery from suicidal ideation. Without longitudinal data on the psychological, behavioural, and social determinants of suicide attempt and the remission of suicidal ideation, we are unlikely to see major gains in the prevention of suicide.

**Aim:**

The LifeTrack Project is a population-based longitudinal cohort study that aims to identify key modifiable risk and protective factors that predict the transition from suicidal ideation to suicide attempt or remission of suicidal ideation. We will assess theory-informed risk and protective factors using validated and efficient measures to identify distinct trajectories reflecting changes in severity of suicidal ideation and transition to suicide attempt over three years.

**Methods:**

A three-year prospective population-based longitudinal cohort study will be conducted with adults from the general Australian population who initially report suicidal ideation (n = 842). Eligibility criteria include recent suicidal ideation (past 30 days), aged 18 years or older, living in Australia and fluent in English. Those with a suicide attempt in past 30 days or who are unable to participate in a long-term study will be excluded. Participants will be asked to complete online assessments related to psychopathology, cognition, psychological factors, social factors, mental health treatment use, and environmental exposures at baseline and every six months during this three-year period. One week of daily measurement bursts (ecological momentary assessments) at yearly intervals will also capture short-term fluctuations in suicidal ideation, perceived burdensomeness, thwarted belongingness, capability for suicide, and distress.

**Conclusion:**

This study is intended to identify potential targets for novel and tailored therapies for people experiencing suicidal ideation and improve targeting of suicide prevention programs. Even modest improvements in current treatments may lead to important reductions in suicide attempts and deaths.

**Study Registration:**

Australian New Zealand Clinical Trials Registry identifier: ACTRN12623000433606.

## Background

More than 700,000 people around the world die by suicide annually [1]. Despite reductions in global age-standardised suicide mortality rates, suicide is a leading cause of age-standardised years of life lost globally [2], and we continue to have limited knowledge of causal factors for the transition from suicidal ideation to suicidal behaviour [3, 4]. In Australia, more than 3,000 people die by suicide annually and suicide is the leading cause of death for those aged 15–44 years [5]. Rates of suicidal ideation (SI) are estimated to be 3.3% per year from national survey data, with 0.3% (i.e., ~ 75,000 Australians annually) reporting a suicide attempt (SA) [6]. Prevalence rates of self-reported SI and SA are even higher in representative Australian cohort studies [7]. Suicide attempts disproportionally occur in younger people, with devastating consequences for family and friends and broader societal, healthcare, and economic costs ($6.73B in Australia) [8].

Despite increased investment in suicide prevention, suicide rates have not declined over recent decades in many nations [6, 9]. This suggests that current clinical interventions for suicidality are not sufficiently effective, possibly because they are poorly targeted. While individuals are encouraged to seek support from general practitioners, hospitals, and mental health professionals, there are limited therapeutic interventions that directly target suicidal thinking and behaviour, with many focusing on reduction of depression symptoms. Suicidality and depression have considerable commonality, with SI representing one symptom of depression in Diagnostic and Statistical Manual of Mental Disorders-5 (DSM-5) and International Classification of Diseases 10th revision (ICD-10) criteria, and common underlying vulnerabilities [10]. However, Cognitive-Behaviour Therapy (CBT)-based interventions that reduce depression symptoms only have a small to medium effect on SI [11], and meta-analytic evidence shows that psychological and pharmacological treatments have only modest efficacy for reducing SI and SA [12]. There are also key differences between the trajectories of suicidality and depression, with remission in SI only partially explained by reductions in depressive symptoms [13]. Among individuals who report SI, suicidal behaviour is more closely tied to post-traumatic stress disorder (PTSD), obsessive-compulsive disorder (OCD), bipolar disorder and conduct disorder than depression [13, 14].

Public health approaches to suicide prevention suggest that multiple strategies are required to reduce suicidal behaviours [15]. However, limited knowledge about the effectiveness of current approaches to prevention and treatment means that a better understanding of the mechanisms underlying suicidal behaviour is needed. In particular, clinical treatments have limited efficacy for reducing suicidal thoughts and attempts. Focusing only on “high risk” patients in clinical settings will always miss a substantial proportion of people who attempt suicide in the community, many of whom are untreated [16]. Crisis services are not equipped to handle the large numbers of individuals who attempt suicide [17], and many people experiencing suicidal distress choose not to engage with health services [18, 19]. Clinical services also have limited resources to provide ongoing support for suicidal individuals. Suicide will only be fully understood and prevented through the inclusion of people in research who do not seek help. Both large scale public health approaches and improved clinical treatments that equip people with the tools to overcome SI and prevent SA are vital to preventing suicide in the community.

A more comprehensive understanding of the modifiable risk factors that predict the transition from SI to SA in adults, including those not in contact with clinical services, is necessary to understand the mechanisms underlying the transition from SI to SA and to inform new treatment and prevention approaches for reducing rates of SA. Theories of suicidal behaviour aim to identify these factors and the mechanisms by which they affect SI and SA. Three recent theories of suicidal behaviour with empirical support are the Interpersonal-Psychological Theory of Suicidal Behaviour (IPTS) [20], the Three-Step Theory (3-ST) [21] and Integrated Motivational-Volitional (IMV) theory [22]. Each theory acknowledges that understanding the key transition from SI to SA is vital for reducing suicide deaths. The IPTS posits that feelings of *thwarted belongingness* (not feeling accepted by others) and *perceived burdensomeness* (a feeling that one is a burden on others) drive the development of SI, but a third factor, the *capability for suicide* (reductions in fear and pain sensitivity sufficient to overcome self-preservation reflexes), is necessary for the transition to SA [23]. However, a systematic review of the theory found very few studies detecting a significant effect of capability for suicide on SA, and those that do find an effect have typically been cross-sectional retrospective studies with small effect sizes [24].

The IMV and 3-ST propose that a broader range of factors, including capability for suicide, access to means, psychopathology, and impulsivity all play key roles. More specifically, the IMV model of suicidal behaviour proposes that defeat and entrapment drive the development of SI and that volitional moderators, such as access to means, exposure to suicidal behaviour, capability for suicide, planning, impulsivity, mental imagery, and past suicidal behaviour, drive the transition from SI to SA [22]. Similarly, the 3-ST proposes that the progression from SI to SA is facilitated by dispositional factors (e.g., pain sensitivity, blood phobia), acquired factors (e.g., habituation to experiences of pain, injury, fear, and death) and practical factors (e.g., knowledge of, and access to, lethal means) [21].

Variability in risk factors may also be relevant to transition from SI to SA. Despite evidence of large short-term variability in suicidal ideation and proposed risk factors of suicidal ideation (e.g., hopelessness and burdensomeness) among psychiatric inpatients and people with a history of suicide attempt/s [25, 26], there is limited understanding of how this variability affects suicide risk over both the short- and long-term in the general population, and therefore a need for prospective burst measurement studies.

There is insufficient high-quality evidence for which factors are most influential in the transition from SI to SA, noting that there is likely to be considerable variability between different population groups. There are no existing population-based longitudinal studies that have adequately explored the role of multiple factors in predicting the transition from SI to SA. Existing studies have relied on retrospective reports of SI/SA [27, 28] or relied on small prospective samples (*n* < 70) [29, 30]. No previous study has been adequately powered to prospectively assess the roles of a comprehensive array of key risk factors for the transition to SA among adults who experience SI. There is also limited evidence around the factors that promote the remission of SI. Previous research suggests that protective factors such as social support and positive mental health are likely to influence a positive course of suicidal thinking [31, 32].

The LifeTrack Project will test the three contemporary theories of SA, as well as novel determinants, to identify the most prevalent pathways from SI to both SA and remission of SI, in the short-term and long-term.

## Method

### Aim

The LifeTrack Project aims to: (a) identify key risk and protective factors that predict the transition from SI to SA, (b) identify distinct trajectories of suicidal ideation severity, remission and/or transition to suicide attempt, and (c) identify subgroups of individuals with suicidal ideation who are most at risk for suicide attempt. We will assess theory-informed risk and protective factors related to psychopathology, cognition, psychological factors, social factors, treatment use, and environmental exposures at repeated intervals, using validated and efficient measures.

Aim 1: Prospectively identify key risk and protective factors that predict the transition from suicidal ideation to suicide attempt, or alternatively from suicidal ideation to remission from suicidal ideation.

- H1: Transition to SA will be significantly predicted by the risk factors proposed by the three theoretical models (IMV, IPTS, 3-ST; see Table 1).
- H2: Beyond the factors central to the three theoretical models, transition will be further predicted by a combination of psychological, cognitive, social, mental health, physical health, treatment, and demographic factors and adverse experiences.
- H3: Factors associated with the transition from SI to SA will be different from those factors associated with persistent SI or remission of SI.
- H4: Greater short-term variability in key constructs related to suicidal behaviour (SI, perceived burdensomeness, thwarted belongingness, and distress, measured in short-term bursts) will be positively associated with transition to SA.

**Table 1:** Assessment domains and instruments

Domain	Instrument	Number of Items	Baseline	6 months / 18 months / 30 months	12 months / 24 months / 36 months	EMA Bursts
**Suicidality**						
Suicide plan / attempt / history	Items adapted from the Youth Risk Behaviour Survey [59]	9	x	x (selected items)	x (selected items)	
Suicidal ideation	Suicidal Ideation Attributes Scale [60]	5	x	x	x	x (selected items)
Self-harm	Items adapted from the Revised Diagnostic Interview for Borderlines [61]	2	x	x	x	
Suicide cognitions	Brief Suicide Cognitions Scale [62]	6	x	x	x	
Methods of coping with suicidality	Open-text question	1	x		x	
**Social/interpersonal**						
Interpersonal needs^*	Interpersonal Needs Questionnaire [63]	10	x	x	x	x (selected bursts)
Exposure to suicide*	Level of Contact Report (suicide) [64]	7	x	x	x	
Fearlessness about death^^#^	Acquired Capability for Suicide Scale – Fearlessness about Death Scale [65]	7	x	x	x	x (selected bursts)
Mental pain tolerance^^#^	Mental Pain Tolerance Scale [66]	10	x	x	x	
Social support*^#^	Schuster Social Support Scale [67]	10	x		x (24 mo only)	
**Mental health**						
Distress thermometer						x
Psychological distress	Distress Questionnaire 5 [68]	5	x	x	x	
Depressive symptoms	Patient Health Questionnaire 9 [69]	9	x		x	
Anxiety symptoms	Generalized Anxiety Disorder 7 [70]	7	x		x	
Social anxiety symptoms	Rapid Management Toolkit: Social Anxiety Disorder [71]	11	x		x	
Obsessive compulsive symptoms	Rapid Management Toolkit: Obsessive Compulsive Disorder [71]		x		x	
Trauma experience and symptoms^#^	Primary Care PTSD Screen for DSM-5 [72]	6	x		x	
Psychotic-like symptoms	PE DISC-IV [73]	6	x		x	
Sleep disturbance	PROMIS sleep disturbance [74]	4	x		x	
Nightmares	Nightmare Experience Scale [75]	4	x		x	
Recovery	Functioning and Recovery Scale [33]	6	x		x	
Wellbeing	WHO-5 Well-Being Index [76]	5	x		x	
Hopelessness^^#^	Brief H Neg [77]	2	x	x	x	
Self-esteem	Brief Rosenberg Self-esteem Scale [78]	5	x	x (18 mo only)		
Future imagery*	Future-oriented items from Impact of Future Events Scale [79]	7	x	x	x	
Impulsivity*	Brief Impulsivity Scale [27]	2	x	x	x	
Emotion processing dysfunction*	Emotional dysregulation subscales of the Emotional Processing Scale [80]	5	x		x (24 mo only)	
Entrapment/defeat*	Short Defeat and Entrapment Scale [81]	8	x		x (24 mo only)	
Agitation	Brief Agitation Measure [82]	3	x		x (24 mo only)	
Perseveration and planning*	Goal Adjustment Scale [83]	10	x		x (24 mo only)	
Discomfort tolerance*	Discomfort Intolerance Scale [84]	7	x		x (24 mo only)	
Rumination*	Brief Response Styles Questionnaire [85]	5	x		x (24 mo only)	
**Other**						
Substance use	Alcohol Use Disorders Identification Test - Consumption [86] + Drug Use Disorders Identification Test - Consumption [87]	7	x		x	
Health	Physical illness checklist [88], self-rated health [89], and medication use	21	x		x	
Pain interference^#^	PROMIS Pain Interference Short Form 4a [90]	4	x			
Treatment use	Actual Help Seeking Scale [91], medication use	13	x		x	
Health-related quality of life	EuroQOL-5D [92]	5	x		x	
Stressful life events	Life Events Checklist [93]	17	x		x	
Work and financial distress	Items from Regional Wellbeing Survey [94]	6	x		x	
Impaired functioning	Work & Social Adjustment Scale [95]	5	x		x	
Sociodemographics	Age, gender identity, sexual orientation, relationship status, education, employment status, ethnicity, rurality, parenting status, smoking/vaping status	10	x	x (selected items)	x (selected items)	

*Factors from the IMV

^#^Factors from the 3-ST

^Factors from the IPTS

Aim 2: Identify distinct trajectories of SI severity, including remission and transition into SA, and the predictors of these classes of trajectories.

- H5: Distinct trajectories (classes) of SI over time will be identifiable statistically, and these trajectories will have different levels of risk for transition to SA.
- H6: Different trajectories across different SI severities will be predicted effectively by baseline factors and short-term variability in key constructs.

Aim 3: Identify subgroups of individuals most at risk of SA.

- H7: Distinct clusters of individuals with differing risk profiles for transition to SA across the follow-up period will be identified.

### Pilot work

In previous qualitative work, we examined factors associated with risk of SA [33, 34]. This research has informed the constructs included in the study that may influence the transition from SI to SA. Population-based quantitative research has also demonstrated preliminary evidence for the roles of a range of specific risk factors in the transition to SA. These include the roles of interpersonal factors [7, 24, 35, 36] and mental disorders including PTSD, OCD, and depression [10, 14, 37], relationship quality/breakdown [38, 39], and psychotic-like experiences [40]. This previous work ensures that the variables included in the study are the most appropriate and relevant for answering the research questions.

This study was funded by the Australian National Health and Medical Research Council (GNT2014841), approved by the Australian National University Human Research Ethics Committee (2022/851), and registered as a longitudinal cohort study with the Australian New Zealand Clinical Trials Registry (identifier: ACTRN12623000433606).

### Design and measures

We will conduct a three-year prospective longitudinal cohort study of adults who report SI at baseline. The follow up period of three years has been chosen to enable sufficient time to establish long-term trajectories and capture relatively rare outcomes including SA. SA will be assessed based on endorsement of the relevant item from either the modified Youth Risk Behaviour Survey or the Suicidal Ideation Attributes Scale, or through report from a participant’s confidant. Table 1 shows a schedule of the survey measures at each assessment point. Measures have been chosen based on the best available scales for assessing the constructs of interest in epidemiological studies [41]. Timing of SA events will be reported by participants and/or confidants.

This study will be conducted entirely online. Participants will initially complete a screening survey to assess eligibility and obtain their contact details and the contact details of a confidant (to facilitate identification of SA or suicide death and welfare checks if required). Informed consent will be obtained from participants at this point. Two days after completing the screening survey, participants will be emailed the baseline assessment, with up to two weeks to complete the survey. Participants will be invited to complete follow-up assessments every six months for the duration of the study, with the final assessment point being three years after completion of the baseline survey. Follow-up assessments will include a subset of the baseline measures, including SI and SA, capturing frequency, severity and timing. The 6-, 18-, and 30-month assessments are estimated to take 15–20 min to complete while the 12-, 24-, and 36-month assessments will take 25–30 min as they cover more risk factors.

At yearly intervals (immediately after the baseline, one-year, and two-year assessments), participants will also be invited to complete one week of two-minute daily measurement bursts (ecological momentary assessments) to capture short-term fluctuations in current SI, perceived burdensomeness, capability for suicide, and distress. Links to the daily surveys will be sent to participants at 12am Australian Eastern Daylight Time or Australian Eastern Standard Time and will remain open for 24 h.

All surveys will be hosted using the online survey platform REDCap [42, 43]. All survey questions will be optional, except for questions that relate to eligibility or to the primary outcomes of the study (suicidal ideation and attempts). All outputs arising from the study will be reported consistent with STROBE guidelines.

### Eligibility criteria

Eligibility criteria for this cohort study include:

(1) Recent or current suicidal ideation (past 30 days).

(2) No suicide attempt in past 30 days.

(3) Capacity to participate in a long-term study.

(4) Aged 18 years or older.

(5) Living in Australia.

(6) Fluent in English.

(7) Willing to provide contact details for self (email address and mobile number) and a confidant (email address).

(8) Access to a device (desktop, laptop, and/or smartphone) and internet connection.

All eligibility criteria will be self-reported by participants. All participants will be encouraged to engage (or continue engaging) with clinical services, and a clinical psychologist will be made available to maximise participant safety and facilitate referral to services, as outlined in the Ethical considerations section below. The clinician will not provide therapeutic services to participants. Participants who are not eligible according to the above criteria or do not consent will also be provided with feedback and information about support services.

### Recruitment, sample size and follow-up

We will recruit from well-established community recruitment sources to maximise the coverage of the study and diversity of the sample: online, social media, primary care settings, and print media.

Many individuals who experience suicidal ideation or behaviours do not present to clinical services [44, 45]. However, previous research has shown that users of popular social media platforms (particularly Facebook and Instagram) who participate in mental health research report high prevalence of suicidal ideation [14]. Social media provides similarly representative samples to other population recruitment methods and is effective and appropriate for recruiting marginalised populations [46, 47]. Additional recruitment using print media advertising will expand the breadth of the sample to include those who do not interact with social media and to maximise ecological validity. Advertisements will be disseminated in locations where people with elevated risk of suicidal thoughts are likely to attend, including primary care clinics. These recruitment methods will result in samples that are diverse in age and reach marginalised populations (e.g., ethnic minorities and people living with mental illness) that are less likely to respond to traditional population recruitment methods [14, 46–48].

Participants will be compensated for their time with e-gift cards of between $25 and $50. The gift card amounts will depend on the length of the survey and the overall time commitment to date. Compensation for the later surveys will be slightly higher than compensation for the earlier surveys in acknowledgement of participants’ greater overall time commitment at the later stages of the research. The data will be screened for ineligible responses before participants are included in the study.

Participants will receive emailed invitations to participate in each six-monthly survey. Email reminders will be sent every four days if a participant has not yet completed the survey, up to a total of three possible reminders, with each follow-up survey staying open for 30 days to maximise completion rates. The study clinical psychologist will contact participants who do not respond to survey invitations via email and/or telephone. If a participant cannot be reached, the study clinical psychologist will contact their confidant to confirm the participant’s welfare and/or determine timing of SA events.

Participants will be considered to have withdrawn if they: (a) email or call the researchers with a withdrawal request, (b) request to withdraw when the clinical psychologist contacts them to follow up on survey completion, or (c) do not commence two consecutive main (i.e., non-EMA) surveys and do not respond to the clinician’s attempts to make contact. No further follow-up attempts will be made at that stage. De-identified data from withdrawn participants will be retained unless they request its deletion.

### Power analysis

Our power calculation is based on detection of the effect of our explanatory variables on transition from SI to SA. We conservatively assume that at least 15% of the sample will attempt suicide at some stage during the follow-up period, based on research findings that up to 20% of people with SI will attempt suicide over 12 months [29, 30, 47]. To have 90% power to detect a moderate standardised effect of d = 0.5 (i.e., effect size at least half a standard deviation from zero) between those who do vs. do not attempt suicide, we require a sample of N = 374. To allow detection of interactions between multiple modifiable factors and have sufficiently narrow confidence intervals around estimates of population preventable fractions (PPF), we have inflated the target sample size by 35% (equivalent to a four-group comparison, rather than simply SA vs. no SA). Further assuming up to 40% attrition at 36 months, we will recruit a sample of N = 842 participants (374 × 1.35 ÷ 0.6). This sample will also be powered to detect up to five latent classes using growth mixture models and latent class analysis to identify subgroups within the sample based on trajectories of SI or baseline characteristics [49] and for machine learning analyses to identify novel combinations of risk factors [50].

### Statistical methods

To identify factors most strongly associated with the transition from SI to SA and remission from SI (H1-H4, H7), statistical analyses will include Cox proportional hazards regression models (time to SA) and zero-inflated negative binomial mixed models (number of SA), accounting for lifetime SA, with suicide deaths treated as right-censored data. Effects will be converted to PPFs based on estimated hazard ratios, combined with prevalence rates taken from external representative data where available (e.g., [51]) or from the cohort. Growth mixture model analyses will classify subgroups of individuals based on their trajectories of SI severity (H5). We will test for both linear and quadratic trajectories using continuous Suicidal Ideation Attributes Scale severity scores. Latent class analyses will differentiate subgroups of individuals reporting SI at baseline (H7). Multinomial logistic regression analyses will then identify factors associated with each of the identified trajectories or latent classes (H6, H7). We will also explore H6 and H7 using machine learning algorithms [52] to identify novel interactions between factors, using a random split (development-validation) approach to classify participants on the basis of SA and on remission from SI.

### Ethical considerations

Based on findings from multiple meta-analyses and systematic reviews [53, 54] and our own research [55], distress attributable to questions about suicide is very rare in community (and clinical) samples, with suicidal participants substantially more likely to report reduced distress and relief at being asked about their experiences, and with no evidence of iatrogenic effects. We have carefully considered the risks of research participation in exacerbating suicidal thinking, but the evidence strongly suggests that even repeated and intensive questions about suicide in high-risk groups are seen by participants as feasible and acceptable, and do not lead to increased distress even in clinical samples [53–56].

All individuals who participate in the study will receive a list of state and national mental health and suicide prevention resources at multiple timepoints throughout the study that they will be encouraged to utilise if they are experiencing distress or mental health concerns. These resources will include access to an online safety planning tool, an approach which has been shown to reduce suicidal behaviour [57].

A clinical psychologist involved in the project will assess risk when safety protocol criteria are met (e.g., if a recent suicide attempt is reported), or if a participant requests a callback, and refer participants to support services. Participants may telephone or email the research team to request support at any time throughout the study. Participant safety will also be supported by requiring participants to provide contact details for themselves (telephone and email address) and a confidant (email address), which will be used to ascertain participant safety if a participant does not respond to the surveys or reminder emails. Participants will be frequently reminded that they are free to withdraw from the study at any time without negative consequences, and to access treatments or services throughout the duration of the study.

## Discussion

This study uses a population health approach to identify new psychological, social, or community targets that may address escalating suicidality. By identifying factors associated with the transition to SA, suicide prevention interventions can be better targeted to individuals in specific high-risk states and tailored to individual risk profiles. There is heterogeneity in trajectories of SI severity [10, 58] and likely to be considerable heterogeneity in the factors that are associated with transition to SA. Treatments that are tailored to individuals with SI, based on patterns of risk factors and trajectories of SI severity, are likely to be more effective. For example, there may be a cluster of people for whom ruminative and obsessive thinking might play an important role in maintenance of SI, leading to SA. For other clusters, interpersonal factors, sleep disturbance, emotional dysregulation, and/or impulsivity may be key precipitants of SA.

A key strength of this study is its 36-month follow-up period, which was chosen to allow sufficient time to establish long-term trajectories and capture relatively rare outcomes including SA, and the inclusion of burst measurements to facilitate a prospective examination of the effects of short-term variability in SI and its risk factors on SA. Other strengths of the protocol include the inclusion of a wide range of potentially relevant factors and the large sample size and appropriate statistical power.

There are limitations associated with online-only long-term studies, including risk of high attrition. The project will use principles of Eysenbach’s Law of Attrition [96]﻿ to mitigate this risk, including advantage (participant reimbursement), compatibility (enabling survey completion using only an internet browser), complexity (using brief screeners), and push factors (use of email reminders, with the option to add SMS reminders if necessary, and feedback on the study results).

Outcomes of this longitudinal study may inform new public health approaches to reduce suicide attempts in the community, with a focus on risk and protective factors. We will identify the factors most strongly associated with remission from suicidal ideation and behaviours, calculating population preventive fractions to assist in prioritising and targeting suicide prevention efforts by policymakers, community organisations, and service delivery organisations. By delivering public health interventions that are targeted to groups of people who are at greatest risk of SA, we can increase the efficiency and effectiveness of public health interventions for delivery in schools, workplaces, community groups, and through the internet.

## Data Availability

Not applicable.

## List of abbreviations

SI: Suicidal ideation
SA: Suicide attempt
CBT: Cognitive Behavioural Therapy
IPTS: Interpersonal-Psychological Theory of Suicidal behaviour
3-ST: Three-Step Theory
IMV: Integrated Motivational-Volitional
STROBE: Strengthening the Reporting of Observational studies in Epidemiology guidelines
NHMRC: National Health and Medical Research Council
